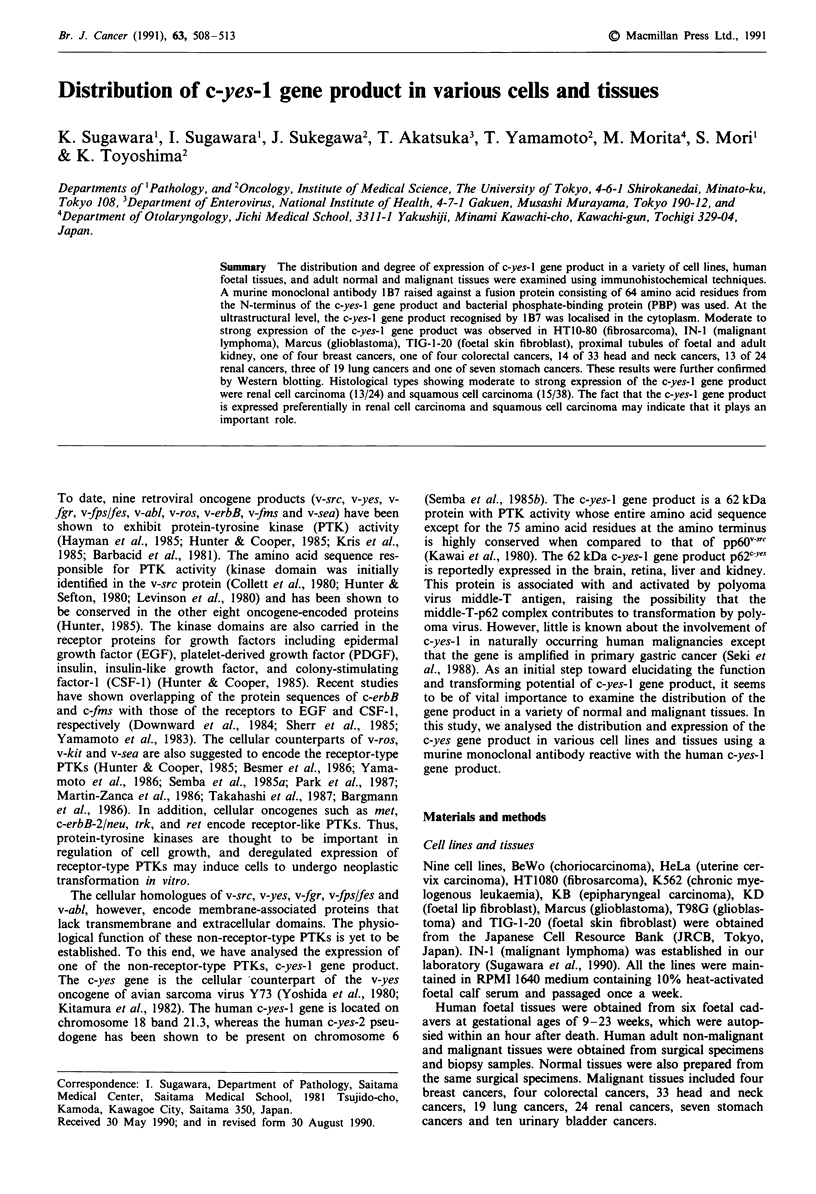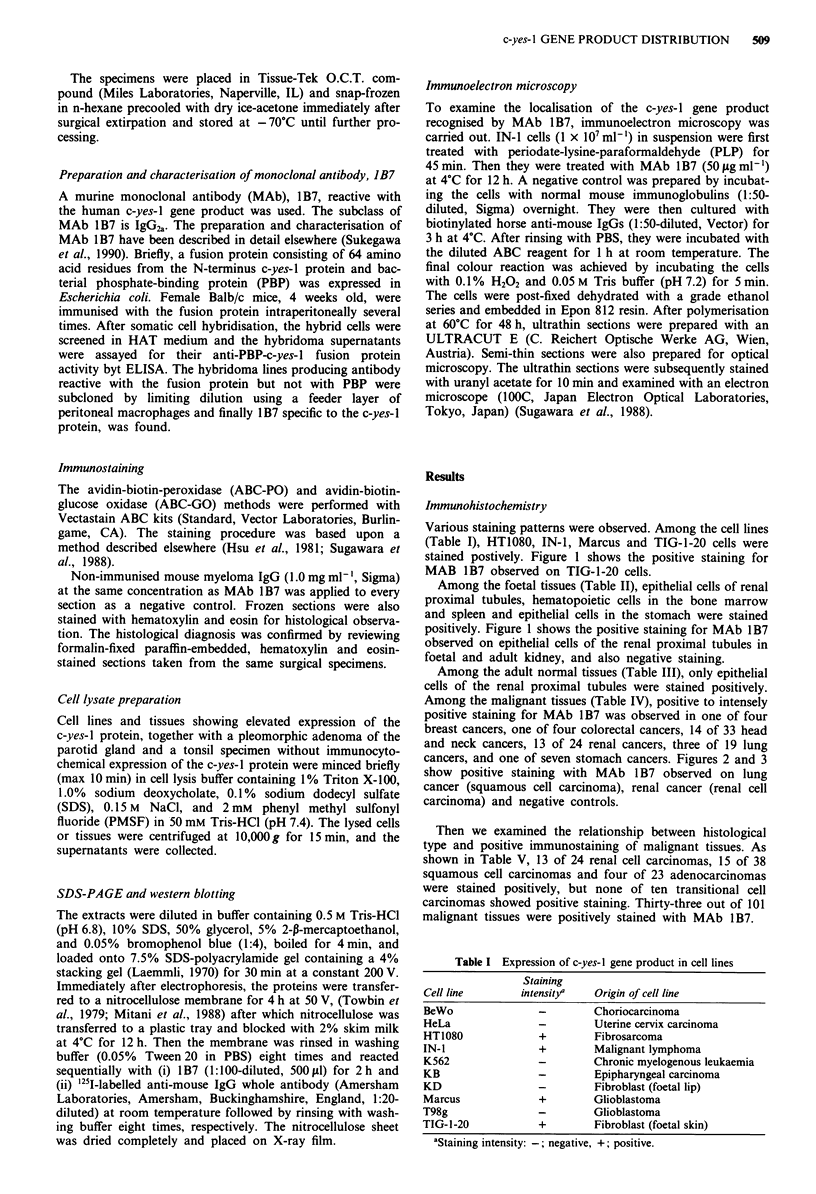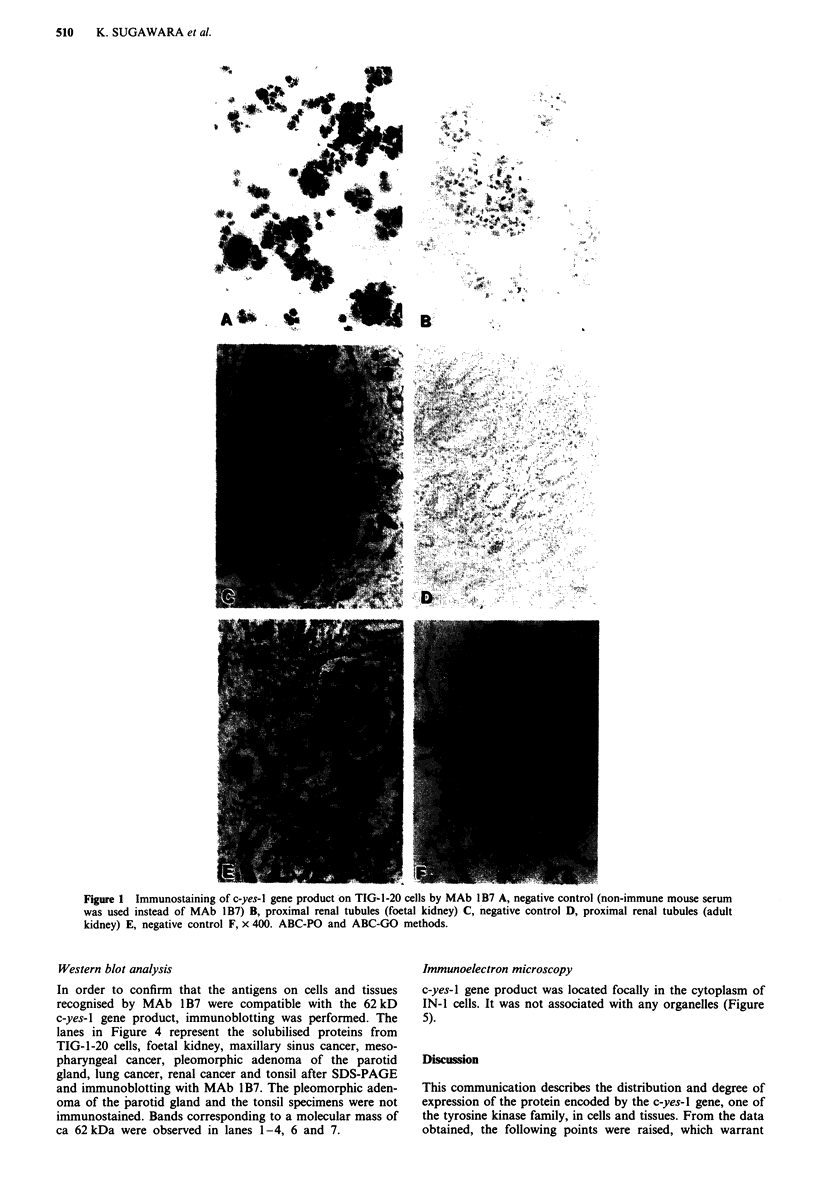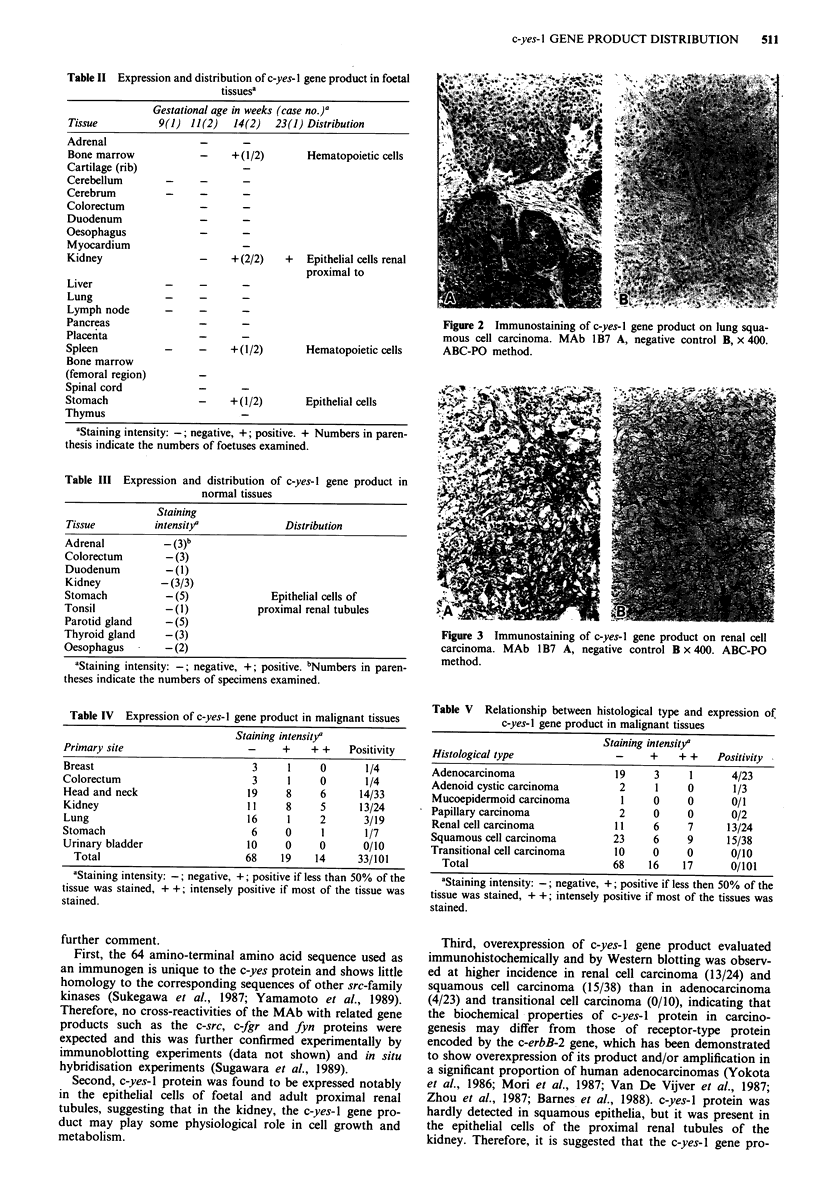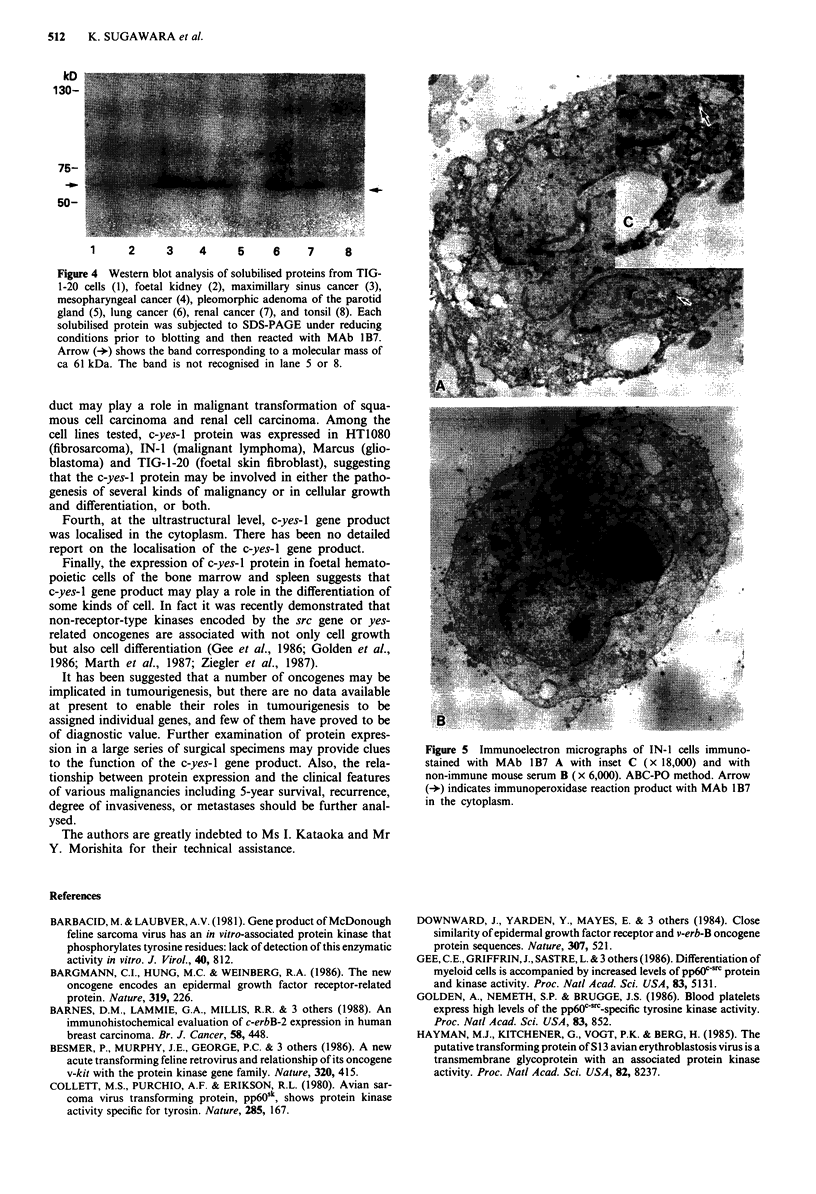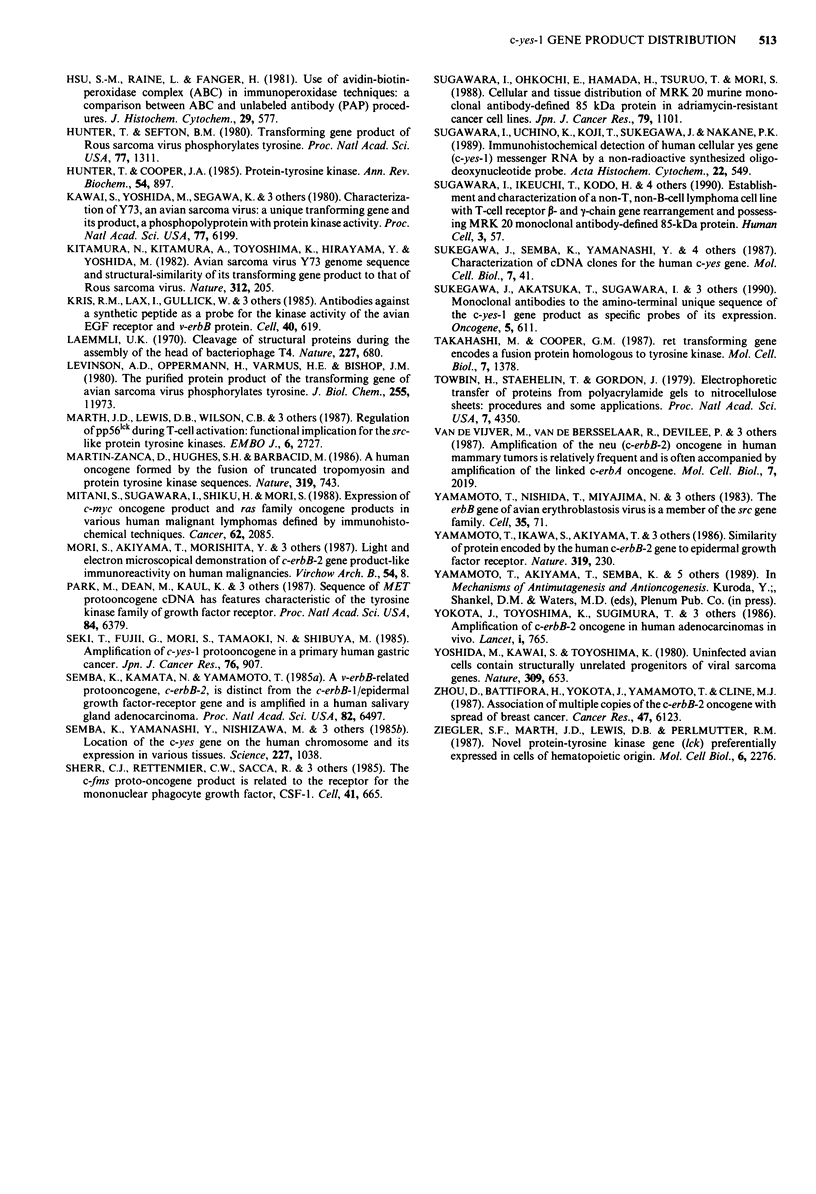# Distribution of c-yes-1 gene product in various cells and tissues.

**DOI:** 10.1038/bjc.1991.121

**Published:** 1991-04

**Authors:** K. Sugawara, I. Sugawara, J. Sukegawa, T. Akatsuka, T. Yamamoto, M. Morita, S. Mori, K. Toyoshima

**Affiliations:** Department of Pathology, University of Tokyo, Japan.

## Abstract

**Images:**


Br. J. Cancer (1991), 63, 508-513 © Macmillan Press Ltd., 1991

### Distribution of c-*yes*-1 gene product in various cells and tissues

K. Sugawara[1], I. Sugawara[1], J. Sukegawa[2], T. Akatsuka[3], T. Yamamoto[2], M. Morita[4], S. Mori[1] & K. Toyoshima[2]

*Departments of [1]Pathology, and [2]Oncology, Institute of Medical Science, The University of Tokyo, 4-6-1 Shirokanedai, Minato-ku, Tokyo 108, [3]Department of Enterovirus, National Institute of Health, 4-7-1 Gakuen, Musashi Murayama, Tokyo 190-12, and [4]Department of Otolaryngology, Jichi Medical School, 3311-1 Yakushiji, Minami Kawachi-cho, Kawachi-gun, Tochigi 329-04, Japan.*

**Summary** The distribution and degree of expression of c-*yes*-1 gene product in a variety of cell lines, human foetal tissues, and adult normal and malignant tissues were examined using immunohistochemical techniques. A murine monoclonal antibody 1B7 raised against a fusion protein consisting of 64 amino acid residues from the N-terminus of the c-*yes*-1 gene product and bacterial phosphate-binding protein (PBP) was used. At the ultrastructural level, the c-*yes*-1 gene product recognised by 1B7 was localised in the cytoplasm. Moderate to strong expression of the c-*yes*-1 gene product was observed in HT10-80 (fibrosarcoma), IN-1 (malignant lymphoma), Marcus (glioblastoma), TIG-1-20 (foetal skin fibroblast), proximal tubules of foetal and adult kidney, one of four breast cancers, one of four colorectal cancers, 14 of 33 head and neck cancers, 13 of 24 renal cancers, three of 19 lung cancers and one of seven stomach cancers. These results were further confirmed by Western blotting. Histological types showing moderate to strong expression of the c-*yes*-1 gene product were renal cell carcinoma (13/24) and squamous cell carcinoma (15/38). The fact that the c-*yes*-1 gene product is expressed preferentially in renal cell carcinoma and squamous cell carcinoma may indicate that it plays an important role.

To date, nine retroviral oncogene products (v-*src*, v-*yes*, v-*fgr*, v-*fps*/*fes*, v-*abl*, v-*ros*, v-*erbB*, v-*fms* and v-*sea*) have been shown to exhibit protein-tyrosine kinase (PTK) activity (Hayman *et al*., 1985; Hunter & Cooper, 1985; Kris *et al*., 1985; Barbacid *et al*., 1981). The amino acid sequence responsible for PTK activity (kinase domain was initially identified in the v-*src* protein (Collett *et al*., 1980; Hunter & Sefton, 1980; Levinson *et al*., 1980) and has been shown to be conserved in the other eight oncogene-encoded proteins (Hunter, 1985). The kinase domains are also carried in the receptor proteins for growth factors including epidermal growth factor (EGF), platelet-derived growth factor (PDGF), insulin, insulin-like growth factor, and colony-stimulating factor-1 (CSF-1) (Hunter & Cooper, 1985). Recent studies have shown overlapping of the protein sequences of c-*erbB* and c-*fms* with those of the receptors to EGF and CSF-1, respectively (Downward *et al*., 1984; Sherr *et al*., 1985; Yamamoto *et al*., 1983). The cellular counterparts of v-*ros*, v-*kit* and v-*sea* are also suggested to encode the receptor-type PTKs (Hunter & Cooper, 1985; Besmer *et al*., 1986; Yamamoto *et al*., 1986; Semba *et al*., 1985*a*; Park *et al*., 1987; Martin-Zanca *et al*., 1986; Takahashi *et al*., 1987; Bargmann *et al*., 1986). In addition, cellular oncogenes such as *met*, c-*erbB*-2/*neu*, *trk*, and *ret* encode receptor-like PTKs. Thus, protein-tyrosine kinases are thought to be important in regulation of cell growth, and deregulated expression of receptor-type PTKs may induce cells to undergo neoplastic transformation *in vitro*.

The cellular homologues of v-*src*, v-*yes*, v-*fgr*, v-*fps*/*fes* and v-*abl*, however, encode membrane-associated proteins that lack transmembrane and extracellular domains. The physiological function of these non-receptor-type PTKs is yet to be established. To this end, we have analysed the expression of one of the non-receptor-type PTKs, c-*yes*-1 gene product. The c-*yes* gene is the cellular counterpart of the v-*yes* oncogene of avian sarcoma virus Y73 (Yoshida *et al*., 1980; Kitamura *et al*., 1982). The human c-*yes*-1 gene is located on chromosome 18 band 21.3, whereas the human c-*yes*-2 pseudogene has been shown to be present on chromosome 6 (Semba *et al*., 1985*b*). The c-*yes*-1 gene product is a 62 kDa protein with PTK activity whose entire amino acid sequence except for the 75 amino acid residues at the amino terminus is highly conserved when compared to that of $pp60^{v\text{-}src}$ (Kawai *et al*., 1980). The 62 kDa c-*yes*-1 gene product $p62^{c\text{-}yes}$ is reportedly expressed in the brain, retina, liver and kidney. This protein is associated with and activated by polyoma virus middle-T antigen, raising the possibility that the middle-T-p62 complex contributes to transformation by polyoma virus. However, little is known about the involvement of c-*yes*-1 in naturally occurring human malignancies except that the gene is amplified in primary gastric cancer (Seki *et al*., 1988). As an initial step toward elucidating the function and transforming potential of c-*yes*-1 gene product, it seems to be of vital importance to examine the distribution of the gene product in a variety of normal and malignant tissues. In this study, we analysed the distribution and expression of the c-*yes* gene product in various cell lines and tissues using a murine monoclonal antibody reactive with the human c-*yes*-1 gene product.

### Materials and methods

### *Cell lines and tissues*

Nine cell lines, BeWo (choriocarcinoma), HeLa (uterine cervix carcinoma), HT1080 (fibrosarcoma), K562 (chronic myelogenous leukaemia), KB (epipharyngeal carcinoma), KD (foetal lip fibroblast), Marcus (glioblastoma), T98G (glioblastoma) and TIG-1-20 (foetal skin fibroblast) were obtained from the Japanese Cell Resource Bank (JRCB, Tokyo, Japan). IN-1 (malignant lymphoma) was established in our laboratory (Sugawara *et al*., 1990). All the lines were maintained in RPMI 1640 medium containing 10% heat-activated foetal calf serum and passaged once a week.

Human foetal tissues were obtained from six foetal cadavers at gestational ages of 9–23 weeks, which were autopsied within an hour after death. Human adult non-malignant and malignant tissues were obtained from surgical specimens and biopsy samples. Normal tissues were also prepared from the same surgical specimens. Malignant tissues included four breast cancers, four colorectal cancers, 33 head and neck cancers, 19 lung cancers, 24 renal cancers, seven stomach cancers and ten urinary bladder cancers.

Correspondence: I. Sugawara, Department of Pathology, Saitama Medical Center, Saitama Medical School, 1981 Tsujido-cho, Kamoda, Kawagoe City, Saitama 350, Japan.
Received 30 May 1990; and in revised form 30 August 1990.

The specimens were placed in Tissue-Tek O.C.T. compound (Miles Laboratories, Naperville, IL) and snap-frozen in n-hexane precooled with dry ice-acetone immediately after surgical extirpation and stored at $-70$°C until further processing.

*Preparation and characterisation of monoclonal antibody, 1B7*

A murine monoclonal antibody (MAb), 1B7, reactive with the human c-*yes*-1 gene product was used. The subclass of MAb 1B7 is $IgG_{2a}$. The preparation and characterisation of MAb 1B7 have been described in detail elsewhere (Sukegawa *et al.*, 1990). Briefly, a fusion protein consisting of 64 amino acid residues from the N-terminus c-*yes*-1 protein and bacterial phosphate-binding protein (PBP) was expressed in *Escherichia coli.* Female Balb/c mice, 4 weeks old, were immunised with the fusion protein intraperitoneally several times. After somatic cell hybridisation, the hybrid cells were screened in HAT medium and the hybridoma supernatants were assayed for their anti-PBP-c-*yes*-1 fusion protein activity byt ELISA. The hybridoma lines producing antibody reactive with the fusion protein but not with PBP were subcloned by limiting dilution using a feeder layer of peritoneal macrophages and finally 1B7 specific to the c-*yes*-1 protein, was found.

*Immunostaining*

The avidin-biotin-peroxidase (ABC-PO) and avidin-biotin-glucose oxidase (ABC-GO) methods were performed with Vectastain ABC kits (Standard, Vector Laboratories, Burlingame, CA). The staining procedure was based upon a method described elsewhere (Hsu *et al.*, 1981; Sugawara *et al.*, 1988).

Non-immunised mouse myeloma IgG (1.0 mg $ml^{-1}$, Sigma) at the same concentration as MAb 1B7 was applied to every section as a negative control. Frozen sections were also stained with hematoxylin and eosin for histological observation. The histological diagnosis was confirmed by reviewing formalin-fixed paraffin-embedded, hematoxylin and eosin-stained sections taken from the same surgical specimens.

*Cell lysate preparation*

Cell lines and tissues showing elevated expression of the c-*yes*-1 protein, together with a pleomorphic adenoma of the parotid gland and a tonsil specimen without immunocytochemical expression of the c-*yes*-1 protein were minced briefly (max 10 min) in cell lysis buffer containing 1% Triton X-100, 1.0% sodium deoxycholate, 0.1% sodium dodecyl sulfate (SDS), 0.15 M NaCl, and 2 mM phenyl methyl sulfonyl fluoride (PMSF) in 50 mM Tris-HCl (pH 7.4). The lysed cells or tissues were centrifuged at 10,000 *g* for 15 min, and the supernatants were collected.

*SDS-PAGE and western blotting*

The extracts were diluted in buffer containing 0.5 M Tris-HCl (pH 6.8), 10% SDS, 50% glycerol, 5% 2-β-mercaptoethanol, and 0.05% bromophenol blue (1:4), boiled for 4 min, and loaded onto 7.5% SDS-polyacrylamide gel containing a 4% stacking gel (Laemmli, 1970) for 30 min at a constant 200 V. Immediately after electrophoresis, the proteins were transferred to a nitrocellulose membrane for 4 h at 50 V, (Towbin *et al.*, 1979; Mitani *et al.*, 1988) after which nitrocellulose was transferred to a plastic tray and blocked with 2% skim milk at 4°C for 12 h. Then the membrane was rinsed in washing buffer (0.05% Tween 20 in PBS) eight times and reacted sequentially with (i) 1B7 (1:100-diluted, 500 μl) for 2 h and (ii) $^{125}$I-labelled anti-mouse IgG whole antibody (Amersham Laboratories, Amersham, Buckinghamshire, England, 1:20-diluted) at room temperature followed by rinsing with washing buffer eight times, respectively. The nitrocellulose sheet was dried completely and placed on X-ray film.

*Immunoelectron microscopy*

To examine the localisation of the c-*yes*-1 gene product recognised by MAb 1B7, immunoelectron microscopy was carried out. IN-1 cells ($1 \times 10^7$ $ml^{-1}$) in suspension were first treated with periodate-lysine-paraformaldehyde (PLP) for 45 min. Then they were treated with MAb 1B7 (50 μg $ml^{-1}$) at 4°C for 12 h. A negative control was prepared by incubating the cells with normal mouse immunoglobulins (1:50-diluted, Sigma) overnight. They were then cultured with biotinylated horse anti-mouse IgGs (1:50-diluted, Vector) for 3 h at 4°C. After rinsing with PBS, they were incubated with the diluted ABC reagent for 1 h at room temperature. The final colour reaction was achieved by incubating the cells with 0.1% $H_2O_2$ and 0.05 M Tris buffer (pH 7.2) for 5 min. The cells were post-fixed dehydrated with a grade ethanol series and embedded in Epon 812 resin. After polymerisation at 60°C for 48 h, ultrathin sections were prepared with an ULTRACUT E (C. Reichert Optische Werke AG, Wien, Austria). Semi-thin sections were also prepared for optical microscopy. The ultrathin sections were subsequently stained with uranyl acetate for 10 min and examined with an electron microscope (100C, Japan Electron Optical Laboratories, Tokyo, Japan) (Sugawara *et al.*, 1988).

### Results

*Immunohistochemistry*

Various staining patterns were observed. Among the cell lines (Table I), HT1080, IN-1, Marcus and TIG-1-20 cells were stained postively. Figure 1 shows the positive staining for MAB 1B7 observed on TIG-1-20 cells.

Among the foetal tissues (Table II), epithelial cells of renal proximal tubules, hematopoietic cells in the bone marrow and spleen and epithelial cells in the stomach were stained positively. Figure 1 shows the positive staining for MAb 1B7 observed on epithelial cells of the renal proximal tubules in foetal and adult kidney, and also negative staining.

Among the adult normal tissues (Table III), only epithelial cells of the renal proximal tubules were stained positively. Among the malignant tissues (Table IV), positive to intensely positive staining for MAb 1B7 was observed in one of four breast cancers, one of four colorectal cancers, 14 of 33 head and neck cancers, 13 of 24 renal cancers, three of 19 lung cancers, and one of seven stomach cancers. Figures 2 and 3 show positive staining with MAb 1B7 observed on lung cancer (squamous cell carcinoma), renal cancer (renal cell carcinoma) and negative controls.

Then we examined the relationship between histological type and positive immunostaining of malignant tissues. As shown in Table V, 13 of 24 renal cell carcinomas, 15 of 38 squamous cell carcinomas and four of 23 adenocarcinomas were stained positively, but none of ten transitional cell carcinomas showed positive staining. Thirty-three out of 101 malignant tissues were positively stained with MAb 1B7.

**Table I** Expression of c-*yes*-1 gene product in cell lines

| *Cell line* | *Staining intensity*[a] | *Origin of cell line* |
|---|---|---|
| BeWo | – | Choriocarcinoma |
| HeLa | – | Uterine cervix carcinoma |
| HT1080 | + | Fibrosarcoma |
| IN-1 | + | Malignant lymphoma |
| K562 | – | Chronic myelogenous leukaemia |
| KB | – | Epipharyngeal carcinoma |
| KD | – | Fibroblast (foetal lip) |
| Marcus | + | Glioblastoma |
| T98g | – | Glioblastoma |
| TIG-1-20 | + | Fibroblast (foetal skin) |

[a]Staining intensity: –; negative, +; positive.

**Figure 1** Immunostaining of c-*yes*-1 gene product on TIG-1-20 cells by MAb 1B7 **A**, negative control (non-immune mouse serum was used instead of MAb 1B7) **B**, proximal renal tubules (foetal kidney) **C**, negative control **D**, proximal renal tubules (adult kidney) **E**, negative control **F**, × 400. ABC-PO and ABC-GO methods.

*Western blot analysis*

In order to confirm that the antigens on cells and tissues recognised by MAb 1B7 were compatible with the 62 kD c-*yes*-1 gene product, immunoblotting was performed. The lanes in Figure 4 represent the solubilised proteins from TIG-1-20 cells, foetal kidney, maxillary sinus cancer, meso-pharyngeal cancer, pleomorphic adenoma of the parotid gland, lung cancer, renal cancer and tonsil after SDS-PAGE and immunoblotting with MAb 1B7. The pleomorphic adenoma of the parotid gland and the tonsil specimens were not immunostained. Bands corresponding to a molecular mass of ca 62 kDa were observed in lanes 1–4, 6 and 7.

*Immunoelectron microscopy*

c-*yes*-1 gene product was located focally in the cytoplasm of IN-1 cells. It was not associated with any organelles (Figure 5).

### Discussion

This communication describes the distribution and degree of expression of the protein encoded by the c-*yes*-1 gene, one of the tyrosine kinase family, in cells and tissues. From the data obtained, the following points were raised, which warrant

**Table II** Expression and distribution of c-*yes*-1 gene product in foetal tissues[a]

| *Tissue* | *Gestational age in weeks (case no.)*[a] 9(1) | 11(2) | 14(2) | 23(1) | *Distribution* |
|---|---|---|---|---|---|
| Adrenal | | – | – | | |
| Bone marrow | | – | +(1/2) | | Hematopoietic cells |
| Cartilage (rib) | | | – | | |
| Cerebellum | – | – | – | | |
| Cerebrum | – | – | – | | |
| Colorectum | | – | – | | |
| Duodenum | | – | – | | |
| Oesophagus | | – | – | | |
| Myocardium | | | – | | |
| Kidney | | – | +(2/2) | + | Epithelial cells renal proximal to |
| Liver | – | – | – | | |
| Lung | – | – | – | | |
| Lymph node | – | – | – | | |
| Pancreas | | – | – | | |
| Placenta | | – | – | | |
| Spleen | – | – | +(1/2) | | Hematopoietic cells |
| Bone marrow (femoral region) | | – | | | |
| Spinal cord | | – | – | | |
| Stomach | | – | +(1/2) | | Epithelial cells |
| Thymus | | | – | | |

[a]Staining intensity: –; negative, +; positive. + Numbers in parenthesis indicate the numbers of foetuses examined.

**Table III** Expression and distribution of c-*yes*-1 gene product in normal tissues

| *Tissue* | *Staining intensity*[a] | *Distribution* |
|---|---|---|
| Adrenal | –(3)[b] | |
| Colorectum | –(3) | |
| Duodenum | –(1) | |
| Kidney | –(3/3) | |
| Stomach | –(5) | Epithelial cells of proximal renal tubules |
| Tonsil | –(1) | |
| Parotid gland | –(5) | |
| Thyroid gland | –(3) | |
| Oesophagus | –(2) | |

[a]Staining intensity: –; negative, +; positive. [b]Numbers in parentheses indicate the numbers of specimens examined.

**Table IV** Expression of c-*yes*-1 gene product in malignant tissues

| *Primary site* | *Staining intensity*[a] – | + | ++ | Positivity |
|---|---|---|---|---|
| Breast | 3 | 1 | 0 | 1/4 |
| Colorectum | 3 | 1 | 0 | 1/4 |
| Head and neck | 19 | 8 | 6 | 14/33 |
| Kidney | 11 | 8 | 5 | 13/24 |
| Lung | 16 | 1 | 2 | 3/19 |
| Stomach | 6 | 0 | 1 | 1/7 |
| Urinary bladder | 10 | 0 | 0 | 0/10 |
| Total | 68 | 19 | 14 | 33/101 |

[a]Staining intensity: –; negative, +; positive if less than 50% of the tissue was stained, ++; intensely positive if most of the tissue was stained.

**Figure 2** Immunostaining of c-*yes*-1 gene product on lung squamous cell carcinoma. MAb 1B7 **A**, negative control **B**, × 400. ABC-PO method.

**Figure 3** Immunostaining of c-*yes*-1 gene product on renal cell carcinoma. MAb 1B7 **A**, negative control **B** × 400. ABC-PO method.

**Table V** Relationship between histological type and expression of c-*yes*-1 gene product in malignant tissues

| *Histological type* | *Staining intensity*[a] – | + | ++ | *Positivity* |
|---|---|---|---|---|
| Adenocarcinoma | 19 | 3 | 1 | 4/23 |
| Adenoid cystic carcinoma | 2 | 1 | 0 | 1/3 |
| Mucoepidermoid carcinoma | 1 | 0 | 0 | 0/1 |
| Papillary carcinoma | 2 | 0 | 0 | 0/2 |
| Renal cell carcinoma | 11 | 6 | 7 | 13/24 |
| Squamous cell carcinoma | 23 | 6 | 9 | 15/38 |
| Transitional cell carcinoma | 10 | 0 | 0 | 0/10 |
| Total | 68 | 16 | 17 | 0/101 |

[a]Staining intensity: –; negative, +; positive if less then 50% of the tissue was stained, ++; intensely positive if most of the tissues was stained.

further comment.

First, the 64 amino-terminal amino acid sequence used as an immunogen is unique to the c-*yes* protein and shows little homology to the corresponding sequences of other *src*-family kinases (Sukegawa *et al.*, 1987; Yamamoto *et al.*, 1989). Therefore, no cross-reactivities of the MAb with related gene products such as the c-*src*, c-*fgr* and *fyn* proteins were expected and this was further confirmed experimentally by immunoblotting experiments (data not shown) and *in situ* hybridisation experiments (Sugawara *et al.*, 1989).

Second, c-*yes*-1 protein was found to be expressed notably in the epithelial cells of foetal and adult proximal renal tubules, suggesting that in the kidney, the c-*yes*-1 gene product may play some physiological role in cell growth and metabolism.

Third, overexpression of c-*yes*-1 gene product evaluated immunohistochemically and by Western blotting was observed at higher incidence in renal cell carcinoma (13/24) and squamous cell carcinoma (15/38) than in adenocarcinoma (4/23) and transitional cell carcinoma (0/10), indicating that the biochemical properties of c-*yes*-1 protein in carcinogenesis may differ from those of receptor-type protein encoded by the c-*erbB*-2 gene, which has been demonstrated to show overexpression of its product and/or amplification in a significant proportion of human adenocarcinomas (Yokota *et al.*, 1986; Mori *et al.*, 1987; Van De Vijver *et al.*, 1987; Zhou *et al.*, 1987; Barnes *et al.*, 1988). c-*yes*-1 protein was hardly detected in squamous epithelia, but it was present in the epithelial cells of the proximal renal tubules of the kidney. Therefore, it is suggested that the c-*yes*-1 gene pro-

**Figure 4** Western blot analysis of solubilised proteins from TIG-1-20 cells (1), foetal kidney (2), maximillary sinus cancer (3), mesopharyngeal cancer (4), pleomorphic adenoma of the parotid gland (5), lung cancer (6), renal cancer (7), and tonsil (8). Each solubilised protein was subjected to SDS-PAGE under reducing conditions prior to blotting and then reacted with MAb 1B7. Arrow (→) shows the band corresponding to a molecular mass of ca 61 kDa. The band is not recognised in lane 5 or 8.

**Figure 5** Immunoelectron micrographs of IN-1 cells immunostained with MAb 1B7 **A** with inset **C** (× 18,000) and with non-immune mouse serum **B** (× 6,000). ABC-PO method. Arrow (→) indicates immunoperoxidase reaction product with MAb 1B7 in the cytoplasm.

duct may play a role in malignant transformation of squamous cell carcinoma and renal cell carcinoma. Among the cell lines tested, c-*yes*-1 protein was expressed in HT1080 (fibrosarcoma), IN-1 (malignant lymphoma), Marcus (glioblastoma) and TIG-1-20 (foetal skin fibroblast), suggesting that the c-*yes*-1 protein may be involved in either the pathogenesis of several kinds of malignancy or in cellular growth and differentiation, or both.

Fourth, at the ultrastructural level, c-*yes*-1 gene product was localised in the cytoplasm. There has been no detailed report on the localisation of the c-*yes*-1 gene product.

Finally, the expression of c-*yes*-1 protein in foetal hematopoietic cells of the bone marrow and spleen suggests that c-*yes*-1 gene product may play a role in the differentiation of some kinds of cell. In fact it was recently demonstrated that non-receptor-type kinases encoded by the *src* gene or *yes*-related oncogenes are associated with not only cell growth but also cell differentiation (Gee *et al.*, 1986; Golden *et al.*, 1986; Marth *et al.*, 1987; Ziegler *et al.*, 1987).

It has been suggested that a number of oncogenes may be implicated in tumourigenesis, but there are no data available at present to enable their roles in tumourigenesis to be assigned individual genes, and few of them have proved to be of diagnostic value. Further examination of protein expression in a large series of surgical specimens may provide clues to the function of the c-*yes*-1 gene product. Also, the relationship between protein expression and the clinical features of various malignancies including 5-year survival, recurrence, degree of invasiveness, or metastases should be further analysed.

The authors are greatly indebted to Ms I. Kataoka and Mr Y. Morishita for their technical assistance.

### References

BARBACID, M. & LAUBVER, A.V. (1981). Gene product of McDonough feline sarcoma virus has an *in vitro*-associated protein kinase that phosphorylates tyrosine residues: lack of detection of this enzymatic activity *in vitro*. *J. Virol.*, **40**, 812.

BARGMANN, C.I., HUNG, M.C. & WEINBERG, R.A. (1986). The new oncogene encodes an epidermal growth factor receptor-related protein. *Nature*, **319**, 226.

BARNES, D.M., LAMMIE, G.A., MILLIS, R.R. & 3 others (1988). An immunohistochemical evaluation of *c-erb*B-2 expression in human breast carcinoma. *Br. J. Cancer*, **58**, 448.

BESMER, P., MURPHY, J.E., GEORGE, P.C. & 3 others (1986). A new acute transforming feline retrovirus and relationship of its oncogene v-*kit* with the protein kinase gene family. *Nature*, **320**, 415.

COLLETT, M.S., PURCHIO, A.F. & ERIKSON, R.L. (1980). Avian sarcoma virus transforming protein, pp60$^{sk}$, shows protein kinase activity specific for tyrosin. *Nature*, **285**, 167.

DOWNWARD, J., YARDEN, Y., MAYES, E. & 3 others (1984). Close similarity of epidermal growth factor receptor and *v-erb*-B oncogene protein sequences. *Nature*, **307**, 521.

GEE, C.E., GRIFFRIN, J., SASTRE, L. & 3 others (1986). Differentiation of myeloid cells is accompanied by increased levels of pp60$^{c\text{-}src}$ protein and kinase activity. *Proc. Natl Acad. Sci. USA*, **83**, 5131.

GOLDEN, A., NEMETH, S.P. & BRUGGE, J.S. (1986). Blood platelets express high levels of the pp60$^{c\text{-}src}$-specific tyrosine kinase activity. *Proc. Natl Acad. Sci. USA*, **83**, 852.

HAYMAN, M.J., KITCHENER, G., VOGT, P.K. & BERG, H. (1985). The putative transforming protein of S13 avian erythroblastosis virus is a transmembrane glycoprotein with an associated protein kinase activity. *Proc. Natl Acad. Sci. USA*, **82**, 8237.

HSU, S.-M., RAINE, L. & FANGER, H. (1981). Use of avidin-biotin-peroxidase complex (ABC) in immunoperoxidase techniques: a comparison between ABC and unlabeled antibody (PAP) procedures. *J. Histochem. Cytochem.*, **29**, 577.

HUNTER, T. & SEFTON, B.M. (1980). Transforming gene product of Rous sarcoma virus phosphorylates tyrosine. *Proc. Natl Acad. Sci. USA*, **77**, 1311.

HUNTER, T. & COOPER, J.A. (1985). Protein-tyrosine kinase. *Ann. Rev. Biochem.*, **54**, 897.

KAWAI, S., YOSHIDA, M., SEGAWA, K. & 3 others (1980). Characterization of Y73, an avian sarcoma virus: a unique tranforming gene and its product, a phosphopolyprotein with protein kinase activity. *Proc. Natl Acad. Sci. USA*, **77**, 6199.

KITAMURA, N., KITAMURA, A., TOYOSHIMA, K., HIRAYAMA, Y. & YOSHIDA, M. (1982). Avian sarcoma virus Y73 genome sequence and structural-similarity of its transforming gene product to that of Rous sarcoma virus. *Nature*, **312**, 205.

KRIS, R.M., LAX, I., GULLICK, W. & 3 others (1985). Antibodies against a synthetic peptide as a probe for the kinase activity of the avian EGF receptor and *v-erbB* protein. *Cell*, **40**, 619.

LAEMMLI, U.K. (1970). Cleavage of structural proteins during the assembly of the head of bacteriophage T4. *Nature*, **227**, 680.

LEVINSON, A.D., OPPERMANN, H., VARMUS, H.E. & BISHOP, J.M. (1980). The purified protein product of the transforming gene of avian sarcoma virus phosphorylates tyrosine. *J. Biol. Chem.*, **255**, 11973.

MARTH, J.D., LEWIS, D.B., WILSON, C.B. & 3 others (1987). Regulation of $pp56^{lck}$ during T-cell activation: functional implication for the *src*-like protein tyrosine kinases. *EMBO J.*, **6**, 2727.

MARTIN-ZANCA, D., HUGHES, S.H. & BARBACID, M. (1986). A human oncogene formed by the fusion of truncated tropomyosin and protein tyrosine kinase sequences. *Nature*, **319**, 743.

MITANI, S., SUGAWARA, I., SHIKU, H. & MORI, S. (1988). Expression of *c-myc* oncogene product and *ras* family oncogene products in various human malignant lymphomas defined by immunohistochemical techniques. *Cancer*, **62**, 2085.

MORI, S., AKIYAMA, T., MORISHITA, Y. & 3 others (1987). Light and electron microscopical demonstration of *c-erbB*-2 gene product-like immunoreactivity on human malignancies. *Virchow Arch. B.*, **54**, 8.

PARK, M., DEAN, M., KAUL, K. & 3 others (1987). Sequence of *MET* protooncogene cDNA has features characteristic of the tyrosine kinase family of growth factor receptor. *Proc. Natl Acad. Sci. USA*, **84**, 6379.

SEKI, T., FUJII, G., MORI, S., TAMAOKI, N. & SHIBUYA, M. (1985). Amplification of *c-yes*-1 protooncogene in a primary human gastric cancer. *Jpn. J. Cancer Res.*, **76**, 907.

SEMBA, K., KAMATA, N. & YAMAMOTO, T. (1985*a*). A *v-erbB*-related protooncogene, *c-erbB-2*, is distinct from the *c-erbB*-1/epidermal growth factor-receptor gene and is amplified in a human salivary gland adenocarcinoma. *Proc. Natl Acad. Sci. USA*, **82**, 6497.

SEMBA, K., YAMANASHI, Y., NISHIZAWA, M. & 3 others (1985*b*). Location of the c-*yes* gene on the human chromosome and its expression in various tissues. *Science*, **227**, 1038.

SHERR, C.J., RETTENMIER, C.W., SACCA, R. & 3 others (1985). The c-*fms* proto-oncogene product is related to the receptor for the mononuclear phagocyte growth factor, CSF-1. *Cell*, **41**, 665.

SUGAWARA, I., OHKOCHI, E., HAMADA, H., TSURUO, T. & MORI, S. (1988). Cellular and tissue distribution of MRK 20 murine monoclonal antibody-defined 85 kDa protein in adriamycin-resistant cancer cell lines. *Jpn. J. Cancer Res.*, **79**, 1101.

SUGAWARA, I., UCHINO, K., KOJI, T., SUKEGAWA, J. & NAKANE, P.K. (1989). Immunohistochemical detection of human cellular yes gene (c-*yes*-1) messenger RNA by a non-radioactive synthesized oligodeoxynucleotide probe. *Acta Histochem. Cytochem.*, **22**, 549.

SUGAWARA, I., IKEUCHI, T., KODO, H. & 4 others (1990). Establishment and characterization of a non-T, non-B-cell lymphoma cell line with T-cell receptor β- and γ-chain gene rearrangement and possessing MRK 20 monoclonal antibody-defined 85-kDa protein. *Human Cell*, **3**, 57.

SUKEGAWA, J., SEMBA, K., YAMANASHI, Y. & 4 others (1987). Characterization of cDNA clones for the human c-*yes* gene. *Mol. Cell. Biol.*, **7**, 41.

SUKEGAWA, J., AKATSUKA, T., SUGAWARA, I. & 3 others (1990). Monoclonal antibodies to the amino-terminal unique sequence of the c-*yes*-1 gene product as specific probes of its expression. *Oncogene*, **5**, 611.

TAKAHASHI, M. & COOPER, G.M. (1987). ret transforming gene encodes a fusion protein homologous to tyrosine kinase. *Mol. Cell. Biol.*, **7**, 1378.

TOWBIN, H., STAEHELIN, T. & GORDON, J. (1979). Electrophoretic transfer of proteins from polyacrylamide gels to nitrocellulose sheets: procedures and some applications. *Proc. Natl Acad. Sci. USA*, **7**, 4350.

VAN DE VIJVER, M., VAN DE BERSSELAAR, R., DEVILEE, P. & 3 others (1987). Amplification of the neu (c-*erbB*-2) oncogene in human mammary tumors is relatively frequent and is often accompanied by amplification of the linked c-*erbA* oncogene. *Mol. Cell. Biol.*, **7**, 2019.

YAMAMOTO, T., NISHIDA, T., MIYAJIMA, N. & 3 others (1983). The *erbB* gene of avian erythroblastosis virus is a member of the *src* gene family. *Cell*, **35**, 71.

YAMAMOTO, T., IKAWA, S., AKIYAMA, T. & 3 others (1986). Similarity of protein encoded by the human c-*erbB*-2 gene to epidermal growth factor receptor. *Nature*. **319**, 230.

YAMAMOTO, T., AKIYAMA, T., SEMBA, K. & 5 others (1989). In *Mechanisms of Antimutagenesis and Antioncogenesis*. Kuroda, Y.;, Shankel, D.M. & Waters, M.D. (eds), Plenum Pub. Co. (in press).

YOKOTA, J., TOYOSHIMA, K., SUGIMURA, T. & 3 others (1986). Amplification of c-*erbB*-2 oncogene in human adenocarcinomas in vivo. *Lancet*, **i**, 765.

YOSHIDA, M., KAWAI, S. & TOYOSHIMA, K. (1980). Uninfected avian cells contain structurally unrelated progenitors of viral sarcoma genes. *Nature*, **309**, 653.

ZHOU, D., BATTIFORA, H., YOKOTA, J., YAMAMOTO, T. & CLINE, M.J. (1987). Association of multiple copies of the c-*erbB*-2 oncogene with spread of breast cancer. *Cancer Res.*, **47**, 6123.

ZIEGLER, S.F., MARTH, J.D., LEWIS, D.B. & PERLMUTTER, R.M. (1987). Novel protein-tyrosine kinase gene (*lck*) preferentially expressed in cells of hematopoietic origin. *Mol. Cell Biol.*, **6**, 2276.